# Sulfonated-Recycled-PEEK as Matrix of Water Vapor Adsorbent SAPO-34 Based Composite Coatings for Adsorption Heat Pumps: Mechanical and Thermochemical Characterization

**DOI:** 10.3390/ma15238439

**Published:** 2022-11-26

**Authors:** Davide Palamara, Luigi Calabrese

**Affiliations:** Department of Engineering, University of Messina, Contrada di Dio Sant’Agata, 98166 Messina, Italy

**Keywords:** sulfonated poly(ether ether ketone), zeolite, coating, adhesion, adsorption heat pump

## Abstract

In this work, a composite adsorbent coating constituted by high SAPO 34 content and a sulfonated recycled poly (ether ether ketone) was investigated for adsorption heat pump technology. Specifically, the effect of polymer recycling on mechanical and thermal properties, as well as on water vapor adsorption and desorption performance, has been investigated. The degree of sulfonation obtained after 48 h of reaction remained approximately unaltered. The degradation of the polymer due to recycling anticipates the degradation of the C-C bonds of the polymer by about 20 °C without affecting the temperature at which the sulfonic groups degrade. From the mechanical point of view, the coating containing 90% zeolite, due to the use of recycled PEEK, evidenced a worsening of only 11.8% in scratch resistance compared to the virgin one, whereas the adhesive strength exhibited an increase of about 23.2% due to better miscibility of the sulfonated recycled polymer. Adsorption/desorption isobars show an almost similar adsorption capacity of the coating produced with recycled polymer compared to the virgin one, confirming that the water vapor diffusion is not hindered by the polymer matrix during the adsorption/desorption process.

## 1. Introduction

Solid adsorption heat transformation (AHT) is a promising, environmental friendly technology alternative to conventional electrically driven systems for heating/cooling production by using low-temperature heat sources (solar, waste heat …) [1].

In this concern, the adsorbent plays a key role in an adsorption heat transformer, significantly affecting the performance of the process [2,3].

The simplest and most economical option is to use an unconsolidated bed in which adsorbent material in the form of granules is placed inside the exchanger fins without a binder. This type of adsorbent bed has a low heat transfer capability due to the punctual contact between the adsorbent grains and the exchanger elements and the low conductivity of the grains themselves [4] but good mass transfer due to the spaces between the grains.

On the other hand, in a consolidated bed the adsorbent material is in direct contact with the metal substrate due either to direct synthesis of the adsorbent material on the substrate [5,6,7] or to the presence of a binder [8,9]. The zeolite-binder coating approach is a capable and affordable technology to apply, in an economical way, an adsorbent layer on the heat exchanger module [10,11,12]. This allows a higher heat transfer at the coating/metal interface, which reduces cycle time [8,13], but a lower mass transfer due to the presence of the binder and the low porosity of the coating.

The low heat and mass transfer in the adsorbent bed are the main causes that make it difficult to achieve high-performance adsorption heat pump (AHP) technology. These factors lead to an increase in the time required to complete adsorption/desorption cycles. From the technological point of view, it is of key importance to find the proper compromise to achieve maximum adsorbate diffusion between the exchanger fins and maximum heat transfer both at the adsorbent/metal interface and between the adsorbent particles themselves. In such a context, in the realization of a binder-based adsorbent composite coating technology, the binder material in addition to having good thermal properties must allow a fast and effective permeation of the water vapor adsorbate.

Initial studies were addressed toward silica gel or zeolite-based coatings involving different inorganic or organic binders as a matrix of the adsorbent composite coatings [14,15]. However, some issues, associated with the hygro-thermal stability and durability of the material, remained unresolved. In this regard, a relevant improvement of knowledge was allowed thanks to Okamoto et al. [16], who assessed performances of a composite coating (constituted by an organic binder as matrix and SAPO-34—coded AQSOA Z02—as filler). Their results evidenced an enhancement of the thermal conductivity of the coated samples (0.36 W/m K) compared to the unconsolidated zeolite bed (0.113 W/m K). Furthermore, zeolite composite coatings constituted of silane-based matrices were investigated and found to have promising adsorption aptitudes, suitable mechanical stability and good durability in severe environmental conditions [17,18]. More recently, Bendix et al. [19] studied several adsorbers differing in the adsorbent/metal ratio. The results evidenced that the heating/cooling power can be optimized, increasing the adsorbent/metal ratio in the HEX. Analogously, Wittstadt et al. [20] investigated a SAPO-34 coated module and confirmed that this technology allows the achieving of a suitable specific power (~82 W/L and ~320 W/L for cooling and heating, respectively) and a coefficient of performance (COP) ~0.4 for cooling and ~1.4 for heating.

However, some drawbacks, related to the limited mechanical resistance and stability of the composite coating (favoring the loss of coating portions from the support during the operating conditions), must be suitably studied. A further issue that requires further investigation is the need to realize coatings with a high zeolite content in order to optimize and tailor the zeolite-based composite coating for AHP applications.

Regarding this concern, Calabrese et al. [21] proposed a new adsorbent composite material, constituted by a siloxane macroporous foam as matrix and SAPO-34 zeolite as adsorbent filler. The results evidenced that the permeable polymer matrix does not hinder the water vapor diffusion [22], providing high mechanical and adsorption performance [23,24].

The choice of using a water vapor-permeable matrix was similarly proposed in [25], where a new adsorbent composite coating constituted by a SAPO-34 powder filled in a sulfonated polyether ether ketone (S-PEEK) matrix is proposed for thermally efficient adsorption heat pumps. The results evidenced that the S-PEEK polymer does not hinder the adsorption/desorption capacity of the SAPO-34 filler, indicating that the proposed technology is promising for practical application in adsorption heat pumps [26,27].

It was also highlighted, from the thermodynamic point of view, that the selection of this suitable coupled adsorbent-matrix allows a cooling COP that is up to over 5% higher than that of the conventional loose adsorbent grains configuration. This suggests this composite coating technology is potentially effective for practical application in adsorption heat pumps.

The same authors subsequently carried out a more in-depth study on the mechanical and physicochemical properties of this zeolite/S-PEEK composite coating, investigating the effect of the degree of sulfonation of the S-PEEK and the zeolite content on the mechanical, thermal and water vapor adsorption properties [28]. The obtained results confirmed that the best compromise between mechanical performance and adsorption capacity was obtained for an intermediate sulfonation of 48 h and a zeolite content of 90 wt.%.

However, considering the costs of the S-PEEK, starting from a virgin raw material, an aspect that can be further considered is the opportunity to have a cheaper raw material that allows the optimization of production costs, preserving the mechanical and adsorption performance of the final composite material.

In such a context, the aim of the present paper is to investigate the use of recycled PEEK as a precursor for producing a sulfonated PEEK matrix for the SAPO-34 composite coating preparation. This choice is able to suitably affect both economic and environmental sustainability of the product. However, it is important that this sustainable choice, at material level, does not affect the adsorbing capacity of the zeolite encapsulated in the S-PEEK matrix.

In particular, in this paper, SAPO-34 zeolite (about 10 μm crystal dimension) was added as filler (in the range 80–95 wt.%) in a sulfonate polyether ether ketone obtained by recycled PEEK (S-rPEEK). The adsorbent composite coating was produced by the drop solvent casting method depositing the composite slurry on an aluminum 6061 metal substrate.

From the experimental characterization point of view, the composite coating was investigated in three steps:

(i) The morphology was evaluated by scanning electron microscopy, in order to evaluate the coating microstructure and homogeneity;
(ii) The chemical-physical and mechanical characterization (this latter performed by scratch and pull-off tests) was also performed to assess the structural stability of the coating and its adhesion with the metal substrate;
(iii) To evaluate the adsorption capacity of the coating under vacuum, at varying water vapor partial pressures, for AHP applications, adsorption equilibrium curves in water vapor of all batches were carried out in the range T = 30–120 °C and pH_2_O = 11 mbar by using a dynamic vapor system, DVS. For comparison, the results referred to S-PEEK-based composite coatings were applied for all tests.

## 2. Materials and Methods

### 2.1. Materials

The recycled polyether ether ketone (PEEK) polymer (glass transition 143 °C, melting point 343 °C) used to realize the composite coating was supplied by Heroflon S.p.A (Collebeato (BS), Italy) in granules of an average size of 0.5–1 mm. The concentrated sulfuric acid (H_2_SO_4_, 98% purity) used to achieve the sulfonation of the recycled PEEK was supplied by Sigma-Aldrich (Burlington, MA, United States). N,N-Dimethyl formamide (N,N-DMF), purity ≥ 99.8%, supplied by Honeywell (Charlotte, NC, United States), was used as a solvent for titration and coating preparation. The adsorbent filler used in the composite coating was a SAPO 34 zeolite (AQSOA Z02), with main grain size of about 5–10 μm, produced by Mitsubishi Plastics Inc. (Tokyo, Japan).

### 2.2. Preparation of S-rPEEK

The sulfonation of the recycled polyether ether ketone polymer was realized by the same procedure reported in [25]. Briefly, 3 g of the recycled PEEK (preliminarily dried at 100 °C for 12 h) were dissolved in 60 mL of concentrated sulfuric acid (98%) at 25 °C under vigorous stirring for 48 h and poured drop by drop into ice-cooled demineralized water in order to rapidly arrest the sulfonation reaction and precipitate the polymer. The acid polymer in the form of small droplets, after an appropriate washing in distilled water in order to obtain the neutralization, was dried at 60 °C for 24 h.

The time of sulfuric acid treatment (48 h) was established as a consequence of the results obtained from the previous work [25] as the best compromise between the mechanical properties obtained and sulfonation times that were not excessive.

For the calculation of the degree of sulfonation (DS) of S-rPEEK, the titration method was used as reported in [29]. Briefly, 0.5 g of S-rPEEK dissolved in 10 mL of N, *N*-Dimethyl formamide was titrated against a standard 0.1 N NaOH solution. Phenolphthalein was used as a virage indicator. *DS* was derived from the following equation:

(1) $$DS=\frac{0.291\cdot M\left(NaOH\right)\cdot V\left(NaOH\right)}{W-0.081\cdot M\left(NaOH\right)\cdot V\left(NaOH\right)}\times 100$$

- *M(NaOH)* is the molarity of the standard NaOH solution (mol/L);
- *V(NaOH)* is the volume of the NaOH solution needed to obtain the virage (mL);
- *W* is the mass of S-PEEK (g);
- 291 and 81 are the molecular weights of the repetitive PEEK unit and the -SO_3_H group, respectively.

### 2.3. Preparation of Composite Zeolite/S-rPEEK Coatings

Four different amounts of S-rPEEK (ranging from 0.2 g to 0.05 g) were dissolved in 1.86 g of N, *N*-Dimethylformamide (DMF). Then, four different amounts of SAPO-34 powder (ranging from 0.8 g to 0.95 g) were added to the polymer solution. The resulting mixture was poured drop by drop on the metal surface (Aluminum 6061) and dried at 60 °C for 12 h. The thicknesses of the resulting coatings were lower than 500 μm. Composite coatings obtained at varying SAPO-34 filler content (80 wt.%, 85 wt.%, 90 wt.% and 95 wt.%) were prepared with the purpose of identifying the optimal compromise between adsorption and mechanical performances.

The four composite S-rPEEK/Zeolite mixtures were coded with a common root “SrP-Z” followed by a number that denotes the dry zeolite content in wt.% employed as filler in the composite coating. For example, SrP-Z80 code refers to a composite coating containing 80 wt.% of SAPO-34 filler in a S-rPEEK (sulfonated recycled PEEK) matrix. Analogously to the recycled one, the virgin-based sulfonated PEEK (S-PEEK), was coded with the prefix “SP-Z” followed by a number indicating the content of SAPO-34 zeolite filler (in wt.%). For example, SP-Z90 refers to the composite coating obtained by adding 90 wt.% of zeolite filler in the S-PEEK polymer obtained starting from virgin PEEK material.

### 2.4. S-rPEEK and Coating Characterization

S-rPEEK thermal behavior was analyzed by thermogravimetric analysis (TGA Q600, TA Instruments, New Castle, DE, USA). Tests were performed in a platinum sample holder under air flow (100 mL/min). Samples (3–5 mg) were preheated at 105 °C for 30 min in order to remove residual moisture; afterwards they were heated, at 5 °C/min, up to 600 °C.

Pull-off and scratch tests were performed to evaluate the mechanical properties of the coatings. The first one, which allows evaluation of the tensile adhesion strength of the coating on the aluminum substrate, was carried out using a DeFelsko PosiTest AT-M pull-off tester (Ogdensburg, NY, USA). The test consists of pulling an aluminum dolly (10 mm in diameter), previously glued on the surface of the coating, by means of an appropriate clamping system. The dolly is glued using a cyanoacrylate adhesive and cured at room temperature for 12 h.

The scratch test, which provides a measure of the scratch resistance of the coating surface (according to EN ISO 1518-1 standard), was performed using an Elcometer 3000 manual Clemen tester (Manchester, UK). A conical-shaped indenter was used (max diameter 2500 μm). It is characterized by a hemispherical AISI 304 stainless steel ball (diameter 500 μm) placed on the top of a truncated conical support (angle 45°, height 1000 μm). The indenter arm is equipped with an adjustable load from 750 g to 2000 g. By dragging the sample along the direction parallel to the arm (at a speed of 10 mm/s), a groove is produced on the surface of the coating by impressing a force proportional to the selected load. Six grooves were made on each sample, under a load increasing from 750 g up to 2000 g. The accuracy in reading the depth of the groove by the 3D optical microscope is related to the ability of the light to be reflected by the surface. This implies that the measurement can be affected by incorrect or excessive illumination of the specimen (the sample is white and the brightness is high). In order to avoid inconsistencies in the measurement, it was preferred to adopt the width of the groove (easier to determine) as a representative parameter of the scratch resistance.

Afterwards, the width and depth of the grooves were measured using a 3D optical digital microscope, HK-8700 (Hirox, Tokyo, Japan).

Finally, by means of a thermogravimetric dynamic vapor system (Surface Measurements Systems DVS Vacuum, London, UK), the isobaric water vapor adsorption/desorption performances of the SAPO-34/S-rPEEK coatings were evaluated. The instrument consists of a micro-balance, with a precision of 0.1 μg, and a water vapor pressure flow control device, inside the measuring chamber, that allows the set pressure to be kept constant. Equilibrium isobars of the composite coatings were measured at 11 mbar. The water uptake was obtained by the following equation:

(2) $$w\left(g/g\right)=\frac{m\left(p_{H_{2}O,}T_{s}\right)-m_{0}}{m_{0}}$$

$m\left(p_{H_{2}O,}T_{s}\right)$ is the sample equilibrium weight at given water vapor pressure and temperature [g];

*m*_0_ is the dried sample weight [g].

Before all measurements, the samples placed in the sample holder were slowly heated up to 150 °C (heating rate 1 °C/min) and kept at this temperature for about 6 h under continuous evacuation (vacuum level: 10^−1^ Pa), to degas the sample and to calculate its dry weight.

For comparison, results referring to unrecycled S-PEEK composite coatings were also reported. Their mechanical and thermo-chemical characterizations are detailed in [25,28].

## 3. Results

### 3.1. Physical-Chemical Characterization and Sulfonation Degree

On synthetized S-rPEEK polymer, preliminarily, the titration method was applied in order to check the degree of sulfonation (*DS*) achieved after acidic treatment. The results, performed on five replicas, evidenced an average degree of sulfonation of 46.52%. This value is comparable with the *DS* of virgin S-PEEK polymer (45.43%) performed by the authors [13] and with the values reported in the literature for the same conditions (48 h of sulfonation, room temperature) [30,31,32]. The compatibility of these data suggests the suitability of the proposed synthesis approach potentially also for recycled polymers.

**Figure 1:**
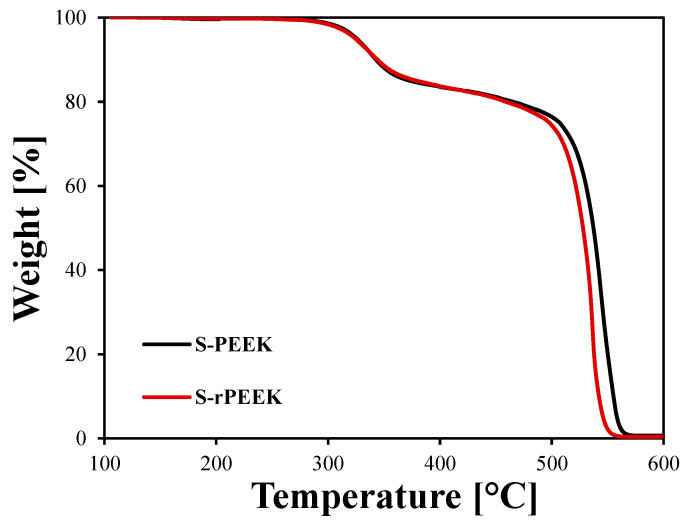
Thermo-gravimetric plot of weight vs. temperature of S-PEEK and S-rPEEK polymer in the range 100–600 °C.

In Figure 1 the thermo-gravimetric curves of S-PEEK and S-rPEEK are plotted. The weight trend at increasing temperature undergoes two abrupt decreases: the first, in a temperature range between 250 and 400 °C, provokes a reduction in weight of about 20%, ascribable to the decomposition of the sulfonic group -SO_3_H [33,34]. A second relevant drop, above 500 °C, is due to the degradation of C-C bonds along the main chain of the polymer [35].

It is noticeable that the two curves are almost similar up to about 500 °C, where the S-rPEEK begins to degrade about 20 °C earlier than the S-PEEK. This indicates that the effect of recycling on the material does not significantly affect its thermal stability, especially considering that the temperature at the sulfonic group degradation, which in S-rPEEK remains unaltered, was given as a temperature threshold suitable for adsorption heat pump applications [17].

**Figure 2:**
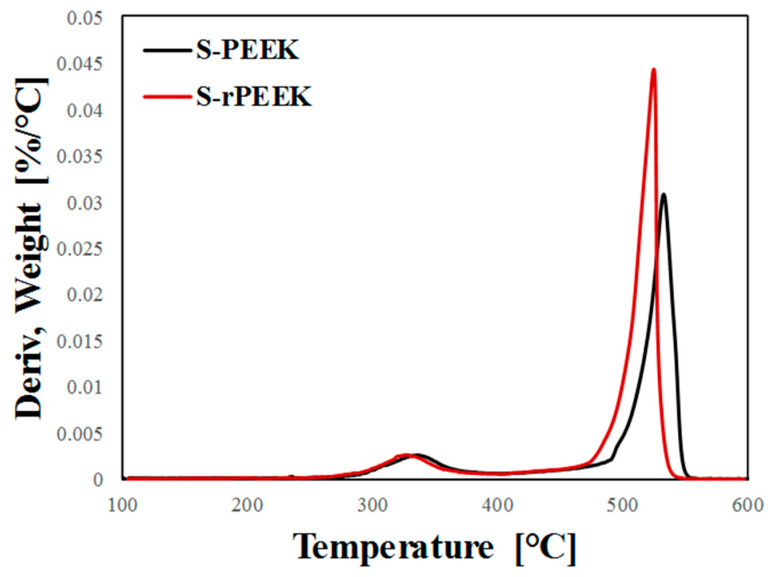
Thermo-gravimetric plot of derivative weight vs. temperature of S-PEEK and S-rPEEK polymer in the range 100–600 °C.

The level of sulfonation that occurred during the process can also be obtained by analyzing the derivative weight-loss curve at increasing temperature (Figure 2). In fact, approximating that the weight loss in the range 250–400 °C is entirely ascribed to the release of -SO_3_, it is possible to estimate the *DS* of S-rPEEK to corroborate the previous results obtained by the titration method. The integral of the peak, at low temperature, in the derivative weight-loss curve is related to the lost mass, allowing the *DS* to be determined according to the following expression [36]:

(3) DS=MPEEK(1A−1)·MSO3H

*A* is the area under the curve in the range 250–400 °C; *M_PEEK_* and *M_SO3H_* are the molecular masses of the PEEK and sulfonic group, equal to 288.7 g/mol and 80.1 g/mol, respectively.

The *DS* values obtained for the two batches, by varying estimation methods, are reported in Table 1.

DS values obtained by thermo-gravimetric analysis show a slightly higher degree of sulfonation in S-rPEEK (48.95%) compared to S-PEEK (46.82%). Analogous considerations can be argued by evaluating the data referring to titration. This could be explained considering that recycled PEEK, due to the applied recycling process, becomes weaker and more easily soluble in sulfuric acid than virgin PEEK. A structurally weaker polymer chain, moreover, allows an easier interaction with sulfonic groups. Thus, the sulfonation process could occur more easily, leading to a slight increase in the DS.

Figure 2 shows that the peak of the weight derivative of S-rPEEK at high temperature, due to polymer chain degradation, is shifted to lower temperature values. Furthermore, the peak of the S-rPEEK polymer is higher and narrower than the virgin matrix peak (S-PEEK), preserving a relatively similar area under the curve. This indicates that the degradation process of the polymer chains is slightly favored using the recycled matrix compared to the virgin one. However, considering the operating conditions in which the coating for heat pump applications operates (<200 °C), this does not imply application constraints.

### 3.2. Mechanical Characterization

It is of primary importance to ensure that the degradation effect due to the recycling of PEEK does not result in relevant reductions in mechanical and adhesive properties at the S-rPEEK/aluminum substrate compared to those obtained with the virgin material. It might be expected that since the mechanical properties of rPEEK are slightly lower than those of PEEK [37], the effect of sulfonation on the recycled PEEK would result in a more pronounced reduction in mechanical properties than that observed in virgin PEEK.

The validation of a good mechanical stability of the absorbent coating is a relevant design parameter because good adhesive and mechanical performance properties of the coating ensure good stability, durability and longevity of AHP systems [38], especially in those application fields (e.g., automotive) that operate under periodic mechanical shocks and vibrations during the service life [39,40]. Therefore, the improvement of mechanical and adhesive performances of the adsorbent material provides relevant information to confirm that the properties of the composite material are suitable to obtain a reliable and durable coated absorber [41]. In this concern, the mechanical adhesive and cohesive properties of the SAPO-34/S-rPEEK composite coating were evaluated by comparing scratch and pull-off tests.

#### 3.2.1. Scratch Tests

The scratch test results were analyzed by comparing the groove widths induced by the indenter tip. Based on the known shape of the indenter, it is possible to identify a direct proportionality between groove depth and width. This choice was made due to the fact that the width, in addition to being easier to measure, is less sensitive to edge effects or local misjudgments due to the influence of the light incidence angle during the optical profile measurement [25]. The width dimension of the notch can be related to the shear strength of the coating, an increase of the width of the notch indicating a reduction in shear strength and a higher sensitivity of the coating to mechanical damage.

**Figure 3:**
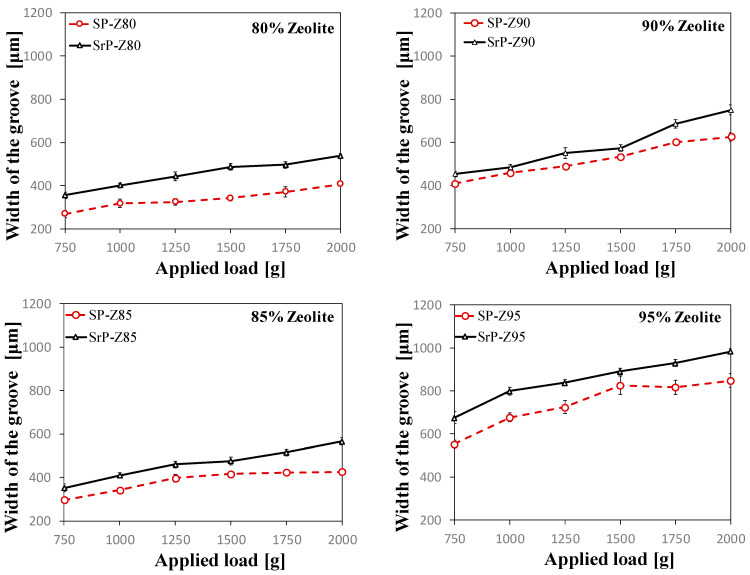
Comparison of groove widths in S-PEEK (SP-Z) and S-rPEEK (SrP-Z) at varying zeolite content.

Figure 3 shows a comparison between the trends of the groove width in the S-PEEK (dashed lines) and S-rPEEK (solid lines) coatings as the applied load increases (from 750 g to 2000 g) at varying zeolite content (between 80 wt.% and 95 wt.%).

It can be observed that the value of the groove width increases as the applied load increases. This trend is quite similar for all composite coating batches (both virgin and recycled matrix). Furthermore, both for S-PEEK and S-rPEEK, the width of the groove increases as the zeolite content increases, indicating a reduction in scratch resistance for batches constituted by high SAPO-34 filler content.

Further evaluating the results reported in Figure 3, it is possible to evidence that the width of S-rPEEK-based coatings is always slightly larger than those of the virgin one (S-PEEK). This effect, identifiable for all formulations, indicates a reduction in scratch resistance due to the reduction in mechanical properties resulting from the recycling of the PEEK. In particular, the mechanical properties of S-rPEEK coatings are about 12–34% lower than S-PEEK one (depending on filler content in the composite coating formulation). This discrepancy becomes less relevant for composite coatings with large zeolite content (85 wt.% and 90 wt%). Analogously to [25], although a progressive decrease in performance is observed for large filler content, the material still preserves a good compromise between good mechanical strength and adsorption capacity. In particular, for SrP-Z90, due to the use of recycled PEEK, a worsening of only 11.8% in scratch resistance was observed.

This trend may be related to the presence of two competing effects, both consequences of PEEK recycling: the worsening of mechanical properties and the increase of miscibility that improves the interaction between filler and matrix. Indeed, for coatings with low zeolite content a large amount of degraded matrix is present, leading to a larger reduction in performances. Increasing the zeolite content decreases the matrix amount, and the worsening of mechanical properties could be mitigated by improved miscibility (ascribable to the slightly higher degree of sulfonation of S-rPEEK compared to virgin one).

**Figure 4:**
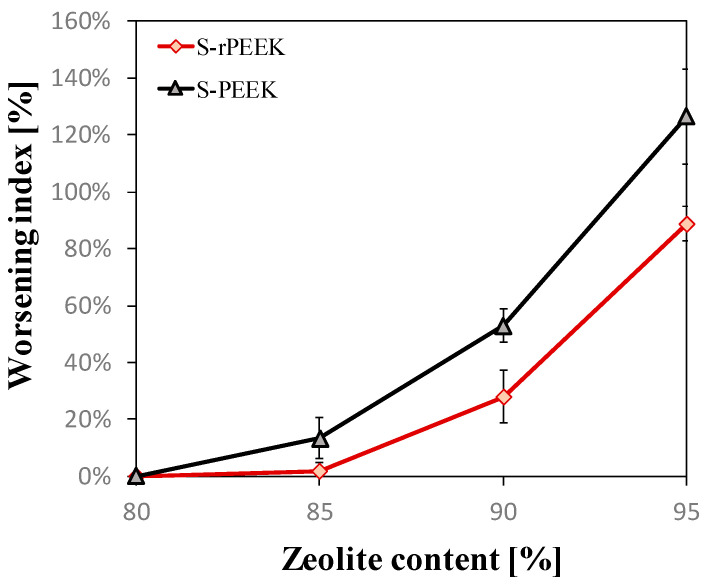
Worsening of scratch resistance properties compared to those obtained from coatings containing 80% zeolite at varying zeolite content for S-PEEK and S-rPEEK.

Figure 4 shows the worsening index (*WI*) of the scratch resistance properties as the zeolite content increases with respect to the properties of the coating containing the lower amount of zeolite (80%). Therefore, the *WI* can be defined as:

(4) $$WI=100\cdot \frac{GW_{i}-GW_{80}}{GW_{80}}$$

where *GW_i_* and *GW*_80_ are the groove width at *i*-th percentage and 80 wt.% of zeolite filler, respectively. In particular, Figure 4 compares the worsening index (determined as average value calculated based on the grooves width obtained from the six different loads for each zeolite content) supplied by S-PEEK and S-rPEEK at increasing filler content.

It can be noticed that in the S-PEEK-based coatings, as the zeolite content increases, a more pronounced worsening of the adhesive properties than S-rPEEK is observed, indicating that there is a more significant increase in groove size than in the S-rPEEK coating.

The performance deterioration as the zeolite content increases can mainly be attributed to the limited amount of matrix, which acts as a binder between the zeolite particles. This implies a reduction of the cohesive force in the constituents of the composite coating. At the same time, the increase in the miscibility of S-rPEEK allows a better interaction between the zeolite particles, at the same matrix content, implying a better mechanical stability of the composite coating for a high content of zeolite filler.

#### 3.2.2. Pull-Off Tests

**Figure 5:**
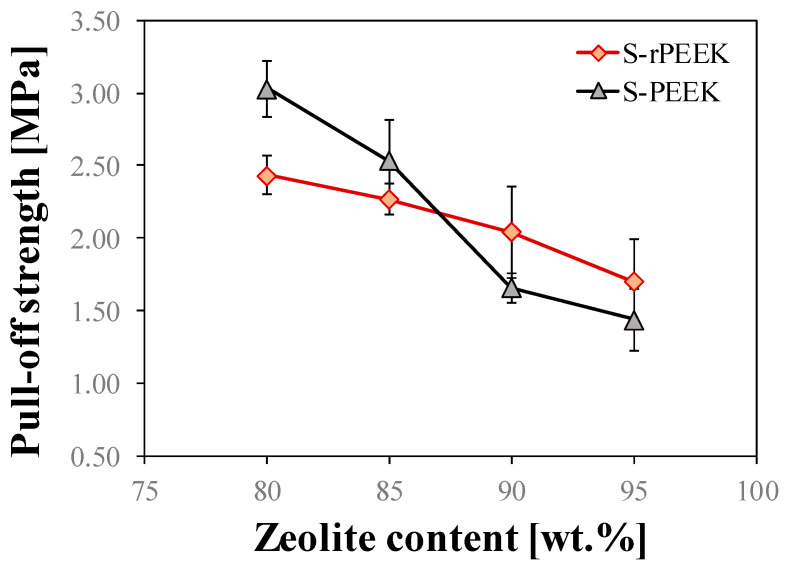
Comparison between pull-off adhesive strength of composite coating at varying filler content of S-PEEK and S-rPEEK.

Figure 5 shows a comparison of pull-off adhesive strength trends in S-PEEK and S-rPEEK composite coatings at increasing zeolite content.

The linear decrease of the adhesive properties with increasing zeolite content confirms that the interactions between filler and matrix constituents within the composite coating play a key role in its mechanical and adhesive properties.

The curve related to the composite coating consisting of a virgin matrix (S-PEEK) has a higher slope than that of the recycled one (S-rPEEK). This result is compatible with what was found for the scratch tests, for which there was found a greater sensitivity of the coating with a virgin matrix to a variation of the mechanical properties as the filler content varies.

It can be observed that initially, in S-rPEEK coatings, the adhesive properties are low. The SrP-Z80 batch showed a pull-off strength 19.7% lower than the SP-Z80 one. However, as the content of zeolite increases, the decrease of the adhesive properties is less relevant, leading the adhesive properties of virgin S-PEEK coating at the highest content of zeolite (90 wt.% and 95 wt.%) to be overcome. In particular, an inversion point takes place at 85–90 wt.%. At highest filler content, SrP-Z95 batch has a pull-off strength about 18.2% higher than the SP-Z95 one.

As previously discussed, this trend may be due to two competing mechanisms: (i) the mechanical properties of recycled PEEK are lower than those of the virgin one, which causes a reduction in adhesive properties in composite coatings constituted by low zeolite content (where a higher matrix amount is present); (ii) the good miscibility of S-rPEEK guarantees a better interaction between the filler constituents. This effect is all the more relevant the higher the filler content (and the lower the matrix content). Consequently, the coatings with recycled matrix perform less well with a low content of zeolite. However, the addition of filler does not involve a significant reduction in the mechanical and adhesive performances, which are also acceptable for zeolite content of up to 95 wt.%.

Nonetheless, all the coatings exhibit higher pull-off strength values than those found in the literature [42,43] confirming a good interaction between filler, matrix and substrate.

In [28], the batch SP-Z90 was indicated as an optimal formulation, considering it as a compromise between high content of zeolite and adequate mechanical performance. It is important to note that the pull-off strength obtained for SrP-Z95 (1.70 ± 0.3 MPa) is slightly higher than that of SP-Z90 (1.66 ± 0.1 MPa). This improvement allows, for this type of matrix, composite coatings with a higher zeolite content, without prejudicing its mechanical stability, with a consequent energy advantage for their use as adsorption heat pumps.

**Figure 6:**
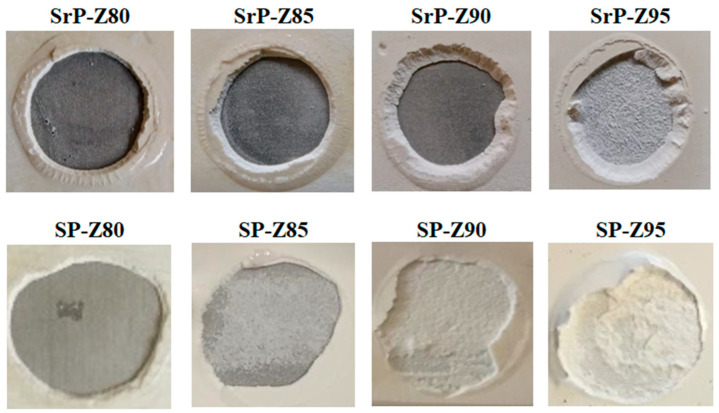
Pull-off fracture surface of the coating after pull-off test in S-PEEK and S-rPEEK.

Further information can be obtained by analyzing the fracture type that occurred during the pull-off test. Figure 6 shows a comparison of the fracture mechanisms of S-PEEK and S-rPEEK at increasing zeolite content.

Conversely to S-PEEK coatings, which show a gradual evolution of the fracture type from totally adhesive to totally cohesive, in S-rPEEK coatings the fracture mechanism changes from adhesive to adhesive/cohesive only at very high zeolite content (from 90 wt.% to 95 wt.% zeolite). When the main fracture mechanism is adhesive (fracture propagates at the coating/metal substrate interface), as it is at low zeolite content, it means that the energy required to trigger a cohesive fracture in the bulk is greater than that required at the coating/substrate interface [44]. When the fracture mechanism is cohesive (fracture propagates into the composite coating bulk), as it is at high zeolite content, the energy required for the crack triggering at the filler/matrix interface is less than that required at the coating/substrate interface, indicating that the filler is not embedded suitably by the matrix.

Particularly, for the SrP-Z95 coating, the fracture is a mixed cohesive/adhesive mode. It propagates at the coating/metal interface involving locally a bulk layer of the composite coating. This fracture mode is in contrast to what occurred in SP-Z95, where the pull-off fracture is dominated by a cohesive mode.

**Figure 7:**
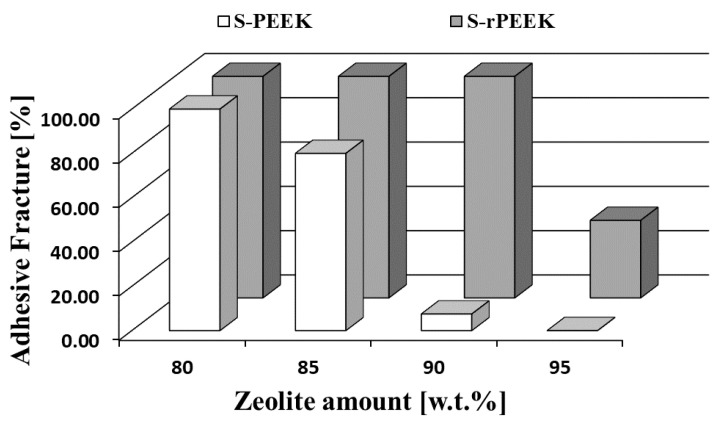
Adhesive fracture mechanism (%) after pull-off test in S-PEEK and S-rPEEK coating at varying filler content.

In fact, the greater cohesive strength of the S-rPEEK coatings compared to the S-PEEK means that, at high zeolite content, a cohesive premature fracture does not occur; a mixed fracture occurs, indicating, indirectly, a greater interaction between filler and matrix in the former formulations. Figure 7 summarizes these finding in a histogram plot where the adhesive fracture mechanisms (expressed in %) observed after the pull-off test in S-PEEK and S-rPEEK coatings at varying filler contents are compared.

### 3.3. Microstructure Characterization

**Figure 8:**
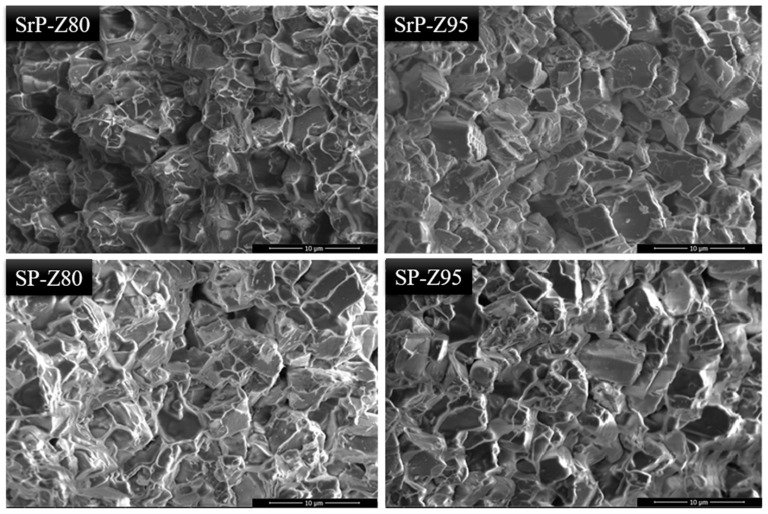
SEM micrographs of fracture surfaces in S-PEEK and S-rPEEK coating at different zeolite content (80 and 95%).

With the purpose of acquiring relevant information on the morphology and homogeneity of the coating microstructure and on the interaction of the matrix with the zeolite particles, surface fracture analyses, obtained in liquid nitrogen (in order to avoid plastic deformation of the matrix) were performed. Figure 8 shows a comparison of SEM micrographs, obtained at 10,000× magnification, of samples containing 80 wt.% and 95 wt.% zeolite filler for both S-rPEEK and reference S-PEEK.

Observing the fracture morphology, it can be confirmed that both matrices interact well with the zeolite grains and present homogeneous mixing without the formation of macro-defects of filler clots. Each zeolite grain is well wrapped in the S-rPEEK and S-PEEK matrices, which can be observed more appreciably in the coatings containing 80 wt.% zeolite (SP-Z80 and SrP-Z80). There are no significant morphological differences between the two coatings. These considerations remain even at high zeolite content (SrP-Z95 and SP-Z95 batches) for which zeolite grains are well interconnected with each other, and the polymeric matrix embeds the solid adsorbent grains in a regular way with no evidence of discontinuity or local detachment.

### 3.4. Adsorption Behaviour

**Figure 9:**
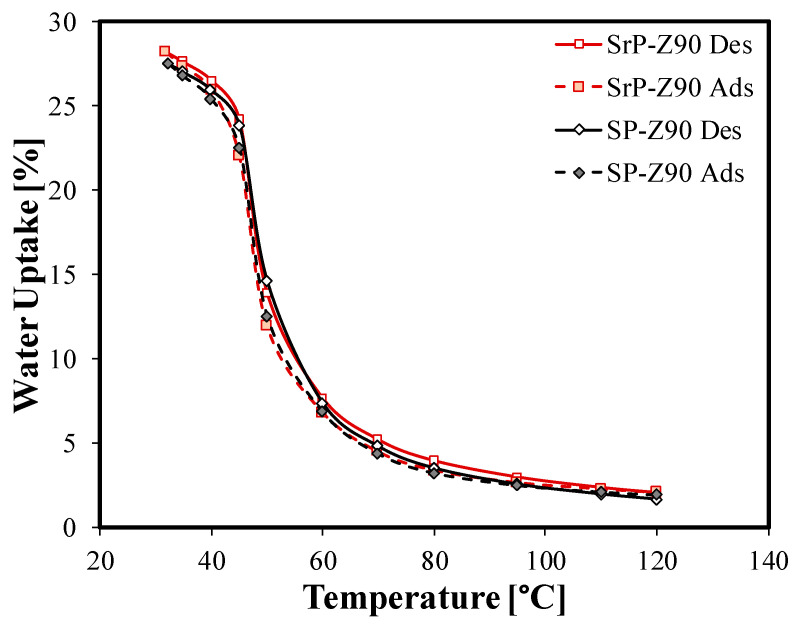
Water adsorption (filled marker) and desorption (empty marker) isobar at 11 mbar for SP-Z90 and SrP-Z90 composite coatings.

In order to evaluate the effective vapor permeability capacity of recycled PEEK and therefore the adsorption/desorption performances, in Figure 9 the adsorption (filled markers) and desorption isobars (empty markers) of the S-PEEK and S-rPEEK composite coatings loaded with 90 wt.% of zeolite are reported. The curves were obtained in the temperature range 30–120 °C at a partial water vapor pressure of 11 mbar. This pressure corresponds to the usual evaporation temperature (8 °C) in an adsorption chiller.

The comparison between the two coatings loaded with 90% of adsorbent material does not show any substantial difference, confirming the fact that S-rPEEK preserves its vapor permeability properties even after recycling. In the range 30–50 °C the water uptake values of S-rPEEK are almost similar to those of the S-PEEK one, confirming the ability of the matrix not to hinder the diffusion of water vapor in the bulk towards the zeolite particles. Moreover the hysteresis of the isobar remains unchanged, thus ensuring that the S-rPEEK-based composite has the same adsorption cycle energy performance as the S-PEEK-based one.

These results allow it to be stated that the composite adsorbent coatings made with a zeolite filler embedded in a recycled S-PEEK polymer matrix can be considered a promising material compared to its virgin counterpart. This potentially poses good prospects to improve this material sustainability, for optimizing the performance of adsorbent beds and maintenance costs in the AHP. Future activities will be aimed at further validating these considerations by enriching the knowledge of their chemical-physical properties (e.g., measurements of thermal conductivity, dynamic adsorption tests, etc.) on a real heat exchanger.

## 4. Conclusions

The effect of the recycling of the PEEK on the performance of composite coatings based on sulfonated recycled PEEK and different amounts of SAPO 34 (80–95 wt.%) was investigated for AHP application. This study has been carried out by analyzing the mechanical and thermal properties as well as the water vapor adsorption and desorption performance. In addition, the coating morphology was studied by SEM microscopy.

Both titration and thermo-gravimetric analysis showed a slight increase in the degree of sulfonation in S-rPEEK at the given treatment duration. The scratch resistance of S-rPEEK coatings is about 10–25% lower than that of the S-PEEK coating. The less pronounced worsening occurs for SrP-Z90, where the difference with SP-Z90 scratch resistance is only 10.4%. In S-rPEEK coatings containing 80% zeolite, the pull-off adhesive strength shows values 19.7% lower than those in S-PEEK. However, in the former, the reduction as the zeolite content increases is less marked, leading to higher adhesion values at higher zeolite content (i.e., 23.2% higher for SrP-Z90). These results can be explained by considering two simultaneous competing effects due to PEEK recycling: the worsening of mechanical properties of the recycled matrix and the increase of miscibility that improves the interaction between filler and matrix.

Morphological analysis exhibits no substantial differences between recycled and virgin polymer, showing that at both high and low zeolite content the filler is well wrapped in the polymer matrix.

Finally, the SrP-Z90 coating showed a slight increase in adsorption capabilities ascribable to the slight increase in the degree of sulfonation due to the recycling of the polymer.

The improvement of the adhesive properties of the coating at high zeolite content could also permit the zeolite content to be increased, allowing further increases in adsorption capabilities of the composite coating without prejudicing its mechanical stability.

These results show that the use of recycled PEEK is a good solution to reduce the manufacturing cost and extend the sustainability of these composite coatings while retaining mechanical and thermal properties and adsorption capacity compatible with AHP applications.

## Figures and Tables

**Table 1:** Values of the degree of sulfonation from titration and the TGA method.

	*DS* by Titration (%)	*DS* by TGA (%)
S-PEEK	45.43 ± 1.95	46.82 ± 2.17
S-rPEEK	46.52 ± 2.11	48.95 ± 2.33

## Data Availability

The data presented in this study are available on request from the corresponding author.
